# The PRC considers military AI ethics: Can autonomy be trusted?

**DOI:** 10.3389/fdata.2022.991392

**Published:** 2022-10-25

**Authors:** Mark Metcalf

**Affiliations:** McIntire School of Commerce, University of Virginia, Charlottesville, VA, United States

**Keywords:** artificial intelligence, intelligentization, lethal autonomous weapons systems, military ethics, People's Liberation Army

## Abstract

China's People's Liberation Army (PLA) is currently wrestling with the benefits and challenges of using artificial intelligence (AI) to enhance their capabilities. Like many other militaries, a key factor in their analysis is identifying and dealing with the ethical implications of employing AI-enabled systems. Unlike other militaries, however, as the PLA is directly controlled by the Chinese Communist Party (CCP—“the Party”), such considerations are conspicuously influenced by a definition of military ethics that is fundamentally political. This Mini-Review briefly discusses key tenets of PLA military ethics and then investigates how the challenges of military AI ethics are being addressed in publicly-available government and PLA publications. Analysis indicates that, while the PLA is considering AI ethical challenges that are common to all militaries (e.g., accountability), their overriding challenge that they face is “squaring the circle” of benefitting from autonomous AI capabilities while providing the CCP with the absolute control of the PLA that it demands—which is, from the CCP's perspective, a military ethics consideration. All Chinese translations are my own.

## Introduction

This paper discusses People's Republic of China (PRC) writings on military artificial intelligence (AI) ethics as they apply to the People's Liberation Army (PLA).

While an abundance of academic and military research about military AI ethics has been published in the PRC, there is currently not a publicly-available *official* PLA policy on the topic.

The PLA is unwilling to publish any materials that may provide potential adversaries insights into their specific considerations and plans for the use of new technologies (such as AI), as such information is considered state secrets. Instead, such writings exhibit a high degree of indirectness. For example, a PLA researcher, instead of unequivocally stating that the PLA should be concerned about the ethical issue of accountability when using lethal autonomous weapons systems (LAWS), will summarize research by *Western* scholars that express concern about the issue. This allows the author to avoid making a policy recommendation—which is the Chinese Communist Party's prerogative—while indirectly highlighting a concern about AI accountability. When evaluating PRC writings on military AI ethics, then, readers must frequently “read between the lines” (relying on multiple sources) to determine what is actually being proposed. They must not only consider what is written, but also how and why specific issues are—and aren't—addressed [(Jullien, 1995), p. 93–115].

As a result, there are currently only a handful of PLA sources that, somewhat authoritatively, express PLA perspectives on military AI ethics and they form the basis of this paper.

## An overview of PLA military ethics

To comprehend the PRC's views regarding military AI ethics, it is important to have a basic understanding how the PLA views the scope and role of military ethics; a perspective that is strongly influenced by the Party (Metcalf, Forthcoming)^1^.

A consistent theme in PLA ethics writings is the importance of developing a “military ethics culture [that] guides soldiers' ethical self-awareness and moral self-discipline” [(Tang, 2016), p. 2]. The PLA considers military ethics to be a political matter that is guided by the Party; an issue that is rooted in Marxist ethics and CCP General Secretary Xi Jinping thought, that supports the development of “socialism with Chinese characteristics”, and contributes to the Party's goal of a creating a strong military as a key element of a rejuvenated China [(Liu and Li, 2020), p. 74]. As a result, PLA writings about military ethics emphasize both the political goals and military benefits of military ethics and rarely engage in discussions of ethics for ethics' sake. In 2017, for example, the Party directed that the PLA should “Follow the Party! Fight to win! Forge exemplary conduct!”—an omnipresent saying in venues ranging from military newspapers to propaganda posters to military facilities. Elsewhere, a PLA political officer explains the strategic significance of military ethics using distinctly political terminology.

The development of military ethics thus embodies the unity of scientific and revolutionary, the unity of theory and practicality. It is not only an important part of the revolutionary change of military culture, but also the historical memory of the nation, a concentrated embodiment of the national spirit with patriotism as the core, and a practical model of the socialist core values. The heroic spirit of the people's army is an important spiritual wealth for self-confidence and the development of socialist culture with Chinese characteristics. The moral practices of the people's army have always played an exemplary and leading role in the process of socialist revolution and construction [(Liu and Li, 2020), p. 74]

PLA ethics training encourages the “cultivation of revolutionary soldiers…having [martial] spirit, having [martial] skills, having courage, and having moral character; the so-called “Four Haves” [(Jia, 2017), p. 4]. The term “spirit” is also used throughout PLA ethics writings to explain the desired characteristics that military ethics are to instill in PLA troops. For example, soldiers are encouraged to follow the spiritual examples of selfless soldiers (e.g., Lei Feng Spirit) and even nationwide political campaigns (e.g., The Resist “SARS” Spirit) [(Liu and Li, 2020), p. 73–74].

PLA military ethics also encourage personnel to conform to socialist and traditional Chinese norms, such as collectivism and selflessness. In recent years, this task has been made difficult due to domestic societal changes and perfidious Western influences. The challenges of turning PRC youth who are increasingly enamored of individuality, making money, or their mobile phones into effective PLA soldiers are frequently mentioned [(Tang, 2016), p. 2–4].

The PLA realizes that the need for ethics training extends beyond merely training personnel to obey the Party. Ethics challenges that are created by emerging technologies (such as AI) must also be addressed. Initially established to tackle the unique roles and responsibilities of PRC defense industry personnel, this topic is also used to address the ethical issues faced by soldiers using ever more capable and lethal weapons systems.

…the relationship between men and weapons are again being developed from a new starting point. The face of warfare is becoming increasingly vague. In modern troop building, military activities, and combat the factor of morality is becoming greater and greater and the matter of military ethics culture is receiving extensive interest. On one hand, the modernization construction of our country's national defense and troops is generating a large number of ethical questions…ethical questions in military training and education, ethical questions in high tech weapons development, ethical questions in military systems, ethical questions regarding military and civilian relationships, knowledge questions regarding the law of war and warfare ethics, etc. They all become questions that must be confronted and settled when reforming a Strong Military. [(Tang, 2016), p. 2–3]

Ethicist Zhao Feng further argues that new and unique ethical issues must be considered as new technologies are developed [(Zhao, 2014), p. 112]. Whether addressing ethical concerns of individuals soldiers or the development of state-of-the-art weapons systems, however, PLA military ethics training consistently emphasizes the incontestable fact that the Party controls the PLA.

## PLA military AI ethics

The PLA is intensely involved in applying AI to their capabilities. Taiwan Army Colonel Jing Yuan-Chou explains that the PLA considers AI to be a “‘game-changing' critical strategic technology; increased machine speed and processing power are expected to be applied to military planning, operational command and decision support as part of the ‘intelligentization' of warfare.” Xi Jinping has directed the PLA to “accelerate the development of military intelligentization,” an endorsement that Jing argues “elevates the concept of intelligentization as a guideline for future Chinese military modernization” (Jing, 2021). The “intelligentization” that Jing describes *specifically* refers to the use of AI to enhance military capabilities and such enhancements result in “intelligentized warfare.”

Given this interest, it may seem surprising that seemingly nothing is available from PLA sources regarding *specific* actions that the PLA is considering to address military AI ethical issues. While this can somewhat be attributed to a PLA penchant for security, there are also political factors resulting from Party control of the PLA. For example, when considering the use of LAWS, at a certain point in the process the PLA operator will “relinquish” control of the weapons system to AI functionality; a procedure that is unacceptable in current PLA doctrine. It is ethical questions like this the PLA must address when considering the use of AI.

### The PRC and military AI ethics

This does not, however, imply that the PRC is absent from international discussions of military AI ethics. At the Sixth Review Conference of the United Nations' Convention on Certain Conventional Weapons in December 2021, the PRC submitted a position paper on the use of military AI which included the following statement on military AI ethics.

In terms of law and ethics, countries need to uphold the common values of humanity, put people's well-being front and center, follow the principle of AI for good, and observe national or regional ethical norms in the development, deployment and use of relevant weapon systems. Countries need to ensure that new weapons and their methods or means of warfare comply with international humanitarian law and other applicable international laws, strive to reduce collateral casualties as well as human and property losses, and prevent misuse and malicious use of relevant weapon systems, as well as indiscriminate effects caused by such behaviors. (MFA-PRC, 2021)

Characterized as being “more aspirational than actionable,” documents like this provide little insight into how the PLA actually views military AI ethics [(Toner, 2022), p. 255–256].

### The PLA and military AI ethics: *PLA Daily*

One type of source that has intermittently shed light on PLA military AI ethics considerations is military newspapers. Vetted by the Party and disseminated throughout the PLA, giving them implicit authority, these sources occasionally provide a forum for regimented discussion of cutting-edge technical or operational issues. For example, a *PLA Daily* article cautions readers about the “ethical black hole of intelligentized warfare”

In the limited practice of intelligentized warfare, the great changes in the style of warfare have raised a series of ethical issues in warfare. In order to correctly understand and handle the relationship between intelligentized warfare and ethics, and to find a balance between technology and human interaction, these ethical issues need to be examined. [(Wu and Qiao, 2020), p. 7]

The authors highlight several ethical issues that are raised by military AI

- The dangers of a virtual battlefield: “Being in the virtual battlefield for a long time may lead to confusion in the judgment of real values, leading to lack of morality and distortion of the concept of war”.
- Inadvertently giving rise to terrorism: While intelligentized weapons systems may improve military operational efficiency and shorten the duration of conflict, “these changes have resulted in a lower threshold for waging war, resulting in frequent violent conflicts, which are contrary to the principles of war ethics” and numb the public to the realities of warfare. In contrast, the side lacking intelligentized systems may have no other recourse than to resort to terrorism in response.
- Attribution of responsibility: “Attribution of responsibility is probably the most criticized ethical issue in intelligentized warfare…Unlike traditional warfare, which can be blamed on specific weapon operators, smart weapons themselves have a certain ability to identify and judge independently. Design flaws, program defects, and operational errors may cause smart weapons to temporarily ‘short-circuit', and responsibility comes naturally. Designers, producers, managers, users and supervisors are required to share the responsibility. This transfer of responsibility has greatly increased the difficulty of assigning responsibility after the war. It also leads to another ethical dilemma—diffusion of responsibility.” [(Wu and Qiao, 2020), p. 7]

The article concludes by stating “technology is a double-edged sword” and bad experiences are an inevitable consequence of development. Much more research will be required before we can “turn intelligentized technology into a technology that is controlled and beneficial to people.” This need to maintain control of AI technology is a frequent theme in PLA writings on the ethical challenges of AI and is consistent with a desire for strict Party control of weapons systems.

A subsequent *PLA Daily* article approached intelligentized weapons systems from a different perspective. A column entitled “In Future Wars, Will ‘Unmanned' Take the Leading Role?” presented different viewpoints on the future implications of unmanned combat [(Liang and Hong, 2021), p. 7]. Liang explained that, throughout history, humans have striven to improve their military capabilities and the intelligentization of weapons systems was yet another step in this process. Eventually, nearly all combat operations would be conducted by unmanned systems and this would result in wars with very few human casualties. Hong rejected this view and argued that human contributions to warfare were essential. From the design of intelligentized systems to the initiation of warfare to the command of combat operations, humans would always be involved.

People are always the equipment controllers and the active factor to bring equipment advantages into play. The more intelligent the weapons and equipment, the more high-level commanders are needed. Therefore, while the battlefield confrontation may be unmanned, combat control must be manned. (Liang and Hong, 2021)

Hong contends that ethical considerations require that “humans are in charge.” Claiming that “military ethics is the moral cornerstone that underpins the modern law of war”, the author recounts the numerous civilian casualties inflicted by US drones in Southwest Asia and concludes

Off-site, non-intuitive, and non-contact implementation of combat operations leads to a lower threshold for war decision-making and a weakening of battlefield moral constraints…Only when humans control the “right to fire” of intelligentized weapons and make unmanned weapons and equipment operate according to human assumptions, can human-machine ethical principles be properly implemented. [(Liang and Hong, 2021), p. 7]

The article concludes by explaining that the two viewpoints highlight the reality that there are still many unanswered questions about intelligentized warfare and that readers should do their best as they work toward developing answers. An interesting aspect of this article is that the two discussants present perspectives that are nearly polar opposites. This would seem to imply that the PLA (and, by extension, the Party) is still wrestling with the operational and ethical implications of incorporating AI into their weapons systems. It is also worth noting that the ethical argument is based on conformity with the law of war and not on any other uniquely-PLA aspects of military ethics. Finally, the article doesn't propose any solutions; only that readers be aware of the challenges and work to solve them.

### The PLA and military AI ethics: Academic journals

A different perspective on military AI systems was presented by AI and intelligentized systems specialists at the PLA Academy of Military Science who highlighted potentially problematic technical, ethical, and strategic AI issues. Ethical issues considered were

- Moral crisis: How should machine rules for unmanned vehicles be established when “the power of choice is decided by the algorithm”?
- Military [security] leaks: Extensive use of commercial AI systems may expose PLA personnel data that reveals military vulnerabilities to hostile forces.
- Military law deficiencies: How will accountability be determined when an intelligentized weapons systems mistakenly destroys civilian targets?
- Development of a subjective consciousness: The danger of “the emergence of a super intelligence that can evolve itself and might develop…into machines controlling society or even enslaving humanity.”
- The emergence of unmanned forces: The threat of one-sided man-vs-machine warfare.

While some of these concerns seem to be more closely tailored to the PLA's perceptions of military ethics (e.g., data breaches affecting combat readiness), each implied that intelligentized systems might result in independent and/or unanticipated operations [(Cai et al., 2019), p. 71–72].

Analyses of foreign research can also provide an awareness of PLA military AI ethics research priorities. Scientists from the National University of Defense Technology conducted PLA-funded research that *specifically* considered the ethical issues associated with LAWS. Interestingly, all of the article's references were from non-PRC publications [(Zhang and Yang, 2021), p. 47]. The authors explained that the fundamental ethical challenges of LAWS are “the dilemma of ‘algorithmic differentiation', the dilemma of military needs and collateral damage, and the responsibility gap caused by the dehumanization of lethal decision-making.” They argued

…the current optimal weapon system is a combination of humans and machines, which not only retains the safety and stability of human judgment, but also takes into account the automation advantages of weapon systems. [(Zhang and Yang, 2021), p. 42]

This “meaningful human control” is consistent with the tenets of PLA military ethics that maintain that the army will fight most effectively while under the direct control of the Party.

## Discussion

While investigating applications of AI in modern warfare the PLA is actively considering ethics, but our understanding of their effort is quite limited due to a paucity of publicly-available information. There is, however, sufficient information to draw the following preliminary conclusions.

### The CCP wants the PLA to implement military AI

Military AI applications offer the promise of new capabilities that could allow the PLA to surpass the capabilities of current and future adversaries. Ignoring AI would put the PLA and the PRC at a strategic disadvantage.

### The PLA is actively investigating the challenges of military AI ethics

While security concerns limit outside access to in-house PLA research, publicly-available materials indicate that PLA analysts are closely monitoring Western military AI ethics research—particularly lessons derived from the Western use of UAVs in Southwest Asia. PLA researchers also understand that military AI will result in significantly challenging ethical considerations and are attempting to resolve such issues.

### PLA discussions of military AI ethics are not political

When discussing military AI ethics, *non*e of the sources discussed Party considerations. Perhaps this is because Party participation is implicit in military ethics discussions, but the absence of political rhetoric is conspicuous by its absence.

### PLA analysis of military AI ethics is highly pragmatic

While PLA authors frequently allude to theoretical aspects of military AI ethics (e.g., accountability, dehumanization, etc.), the general trend of the discussions devolve to the highly practical problem of controlling a system that is, by definition, autonomous. This is an important factor when considering military AI ethics because, from a PLA perspective, appropriate ethical behavior is the logical result of following the Party's guidance.

### The PLA needs to resolve the issue of “autonomy or control” for its AI weapons systems

In the near term, the PLA will continue to employ AI to enhance existing military capabilities, but not to implement fully autonomous systems. This does not mean, however, that the PLA is not considering the use of LAWS. The PLA, like all militaries, wants its forces to be equipped with state-of-the-art capabilities and is undoubtedly actively conducting LAWS research and development. Once the PLA is able to solve this challenge to their satisfaction, and in spite of public declarations to the contrary, it would be surprising if they didn't add such cutting-edge capabilities to the PLA's arsenal.

Given the limited availability of relevant data, future insights regarding PLA military AI ethics developments must continue to be meticulously gleaned and interpreted from authoritative PLA journals and official media—particularly since the PLA and Party are apparently still wrestling with such policies and are disinclined to publicly discuss their deliberations. While exchanges between PRC and non-PRC military ethics specialists could provide additional insights, given the current international political climate, prospects for such interactions seem unlikely.

## Author contributions

MM conceived of and designed the study, translated and analyzed the Chinese language materials, and wrote all sections of the manuscript.

## Conflict of interest

The author declares that the research was conducted in the absence of any commercial or financial relationships that could be construed as a potential conflict of interest.

## Publisher's note

All claims expressed in this article are solely those of the authors and do not necessarily represent those of their affiliated organizations, or those of the publisher, the editors and the reviewers. Any product that may be evaluated in this article, or claim that may be made by its manufacturer, is not guaranteed or endorsed by the publisher.

## Footnotes

^1^Metcalf, M. Forthcoming. “A survey of 21st century PLA scholarship on the role of military ethics in warfare,” in *Warfare Ethics in Comparative Perspective: China and the West*, eds S. B. Twiss, P.-C. Lo, and S. B. Chan (London: Routledge).
